# Supplementary material for: Behavioural analysis of postnatal physical activity in the UK according to the COM-B model: a multi-methods study
Source: BMJ Open. 2019 Aug 2;9(8):e028682. doi: 10.1136/bmjopen-2018-028682 (PMC6686995; doi:10.1136/bmjopen-2018-028682)
Supplement: Supplementary file 2 [file bmjopen-2018-028682supp002.pdf]

Capability

1. **Have you been able to be active recently?**
    - *If no – Why not?*
    - *If yes – can you tell me more about how you were able to do this?*
2. **What do you think makes (would make) it difficult or more difficult to participate in physical activity?**
    - *Can you think of anything that makes it difficult to take part in physical activity?*
3. **What do you think makes (would make) it easy or easier for you to take part in physical activity?**
    - *Can you think of anything that would make it easier for you to take part in physical activity regularly?*

Opportunity

4. **Think about the environment around you, how does this support you to be active?**
    - *How do the local facilities and your local neighbourhood help you to be active?*
    - *If no response, could say: for example, is the local neighbourhood well maintained and make you feel safe, or are the facilities suitable for postnatal women?*
5. **Once again think about the environment around you, how does this make it difficult to be active?**
    - *Can you think of any aspects of the local environment or facilities which make it difficult to be active?*
6. **Are there particular individuals or groups of people that would support or approve you to be physically active?**
    - *Is there anyone you know that would encourage you to be active?*
    - *If no response: for example, is there anyone that would offer to join in with you, or look after your child, or offer you a lot of encouragement?*
7. **Are there particular individuals or groups of people that would disapprove of you being physically active?**
    - *Is there anyone you can think of that would be particularly discouraging?*

Motivation

8. **What do you think are the advantages of participating in physical activity?**
    - *How do you think being active would help you?*
9. **What do you think are the disadvantages of participating in physical activity?**
    - *Is there anything that you can think of that would be worse because you were active?*
10. **What would/does motivate you to be active?**
    - *What are the main reasons that you would want to become active?*

If necessary, the researcher will redefine physical activity to broaden the range of activities that an individual is thinking about to include examples such as walking, gardening, housework, cycling. Researcher will repeat the key points raised during the discussion and ask the following question:

11. **Given that I've now given you examples of more physical activities, does this change any of the points that you have raised?**
